# Cross-cultural adaptation and validation of the teamwork climate scale

**DOI:** 10.1590/S1518-8787.2016050006484

**Published:** 2016-08-16

**Authors:** Mariana Charantola Silva, Marina Peduzzi, Carine Teles Sangaleti, Dirceu da Silva, Heloise Fernandes Agreli, Michael A West, Neil R Anderson

**Affiliations:** I Prefeitura Municipal de Campinas. Secretária de Saúde. Campinas, SP, Brasil; II Departamento de Orientação Profissional. Escola de Enfermagem. Universidade de São Paulo. São Paulo, SP, Brasil; III Departamento de Enfermagem. Universidade Estadual Centro Oeste. Guarapuava, PR, Brasil; IV Departamento Educação. Faculdade de Educação. Universidade Estadual de Campinas. Campinas, SP, Brasil; V Programa de Pós-Graduação em Gerenciamento em Enfermagem. Escola de Enfermagem. Universidade de São Paulo. São Paulo, SP, Brasil; VI Centre for Performance Led HR. Management School. Lancaster University. Lancaster, United Kingdom; VII Brunel Business School. Brunel University. London, United Kingdom

**Keywords:** Scales, Translations, Patient Care Team, organization & administration, Personnel Management, Interpersonal Relations, Validation Studies, Reproducibility of Results

## Abstract

**OBJECTIVE:**

To adapt and validate the Team Climate Inventory scale, of teamwork climate measurement, for the Portuguese language, in the context of primary health care in Brazil.

**METHODS:**

Methodological study with quantitative approach of cross-cultural adaptation (translation, back-translation, synthesis, expert committee, and pretest) and validation with 497 employees from 72 teams of the Family Health Strategy in the city of Campinas, SP, Southeastern Brazil. We verified reliability by the Cronbach’s alpha, construct validity by the confirmatory factor analysis with SmartPLS software, and correlation by the job satisfaction scale.

**RESULTS:**

We problematized the overlap of items 9, 11, and 12 of the “participation in the team” factor and the “team goals” factor regarding its definition. The validation showed no overlapping of items and the reliability ranged from 0.92 to 0.93. The confirmatory factor analysis indicated suitability of the proposed model with distribution of the 38 items in the four factors. The correlation between teamwork climate and job satisfaction was significant.

**CONCLUSIONS:**

The version of the scale in Brazilian Portuguese was validated and can be used in the context of primary health care in the Country, constituting an adequate tool for the assessment and diagnosis of teamwork.

## RESUMO

**OBJETIVO:**

Adaptar e validar a escala *Team Climate Invetory*, de medida do clima de trabalho em equipe, para o idioma português, no contexto da atenção primária à saúde no Brasil.

**MÉTODOS:**

Estudo metodológico com abordagem quantitativa de adaptação transcultural (tradução, retrotradução, síntese, comitê de especialistas e pré-teste) e validação com 497 trabalhadores de 72 equipes da Estratégia Saúde da Família no município de Campinas, São Paulo. Verificou-se confiabilidade pelo alfa de Cronbach, validade de construto pela análise fatorial confirmatória pelo *software* SmartPLS e correlação com escala de satisfação no trabalho.

**RESULTADOS:**

Foi problematizado a sobreposição dos itens 9, 11 e 12 do fator participação na equipe e o fator objetivos da equipe no tocante à sua definição. A validação mostrou que não houve sobreposição dos itens e a confiabilidade variou entre 0,92 a 0,93. A análise fatorial confirmatória indicou adequação do modelo proposto com distribuição dos 38 itens nos quatro fatores. A correlação entre clima de trabalho em equipe e satisfação no trabalho foi significativa.

**CONCLUSÕES:**

A versão da escala em português falado no Brasil foi validada e pode ser utilizada no contexto da atenção primária à saúde no País, constituindo ferramenta adequada para a avaliação e diagnóstico do trabalho em equipe.

## INTRODUCTION

This article presents the process of cross-cultural adaptation and validation of the Team Climate Inventory (TCI) scale in the context of primary health care in Brazil. It provides an instrument that allows the evaluation of teams to monitor the effectiveness of teamwork in producing results in the care of users and population. It also allows distinguishing between more and less effective teams and implementing permanent education actions.

Both in the global^12^ and national^9^ contexts, primary, comprehensive, and integral health care is the most efficient way of tackling the problems of public health and the fragmentation of health systems. In Brazil, the Brazilian Unified Health System (SUS) extends the primary care from the Family Health Strategy (FHS) to the reorganization of the system based on the work of family health teams.

The literature on teamwork highlights key elements that characterize it: communication^10^, collaboration and patient-centered care^3^, shared definition of the team goals^19^, and innovation to answer the health needs of users, families, and community^9^ ^,^ ^19^.

The health teams differ from each other. An aggregation team is characterized by fragmented and juxtaposed actions from different professionals, but the integration team articulates the actions by the interaction of its members^14^. Real teams are groups of people who work together with interdependence and commitment in sharing and achieving common goals. Pseudo-teams are marked by individual work, with little interaction, weak information sharing, and lack of clarity in the definition of common goals to the team^19^. Potential teams identify the need to improve their performance, but with lack of clarity of the role of the members and their common goals. Finally, high-performance teams are characterized by commitment, clarity of roles, and motivation of members^8^. Thus, it is essential to distinguish them as to their effectiveness and impact on the quality of health care.

Considering the complexity of the objects of intervention of the work processes in health care, particularly in the context of primary care, we need instruments that encourage the quick evaluation of teamwork and its capacity for change and innovation according to the health needs of users and the population.

The national literature on evaluation of teamwork shows no instruments that measure this practice in Brazil. On the other hand, the international literature offers tools^5^ ^,^ ^18^, notably the TCI^1^, which is an instrument validated in 11 countries in the business, hospital, and primary health care context, with good psychometric properties and conceptual support. Some studies that applied the TCI to assess primary care teams showed the influence of the organizational climate and culture as predictors for changes and improvement of the quality of teamwork^7^ ^,^ ^15^ and also on the job satisfaction^15^, although they point the need for further studies to confirm this.

TCI is prepared from the concept of organizational climate based on shared perceptions among professionals about policies, practices, and processes in the work environment^1^. The theoretical framework adopted in the TCI corresponds to the understanding of teamwork found in the studies on the topic developed in the Country, especially in the SUS public policy scenario, i.e., on joint actions and interaction between professionals, with emphasis on communication^4^ ^,^ ^11^.

## METHODS

**Figure 1:**
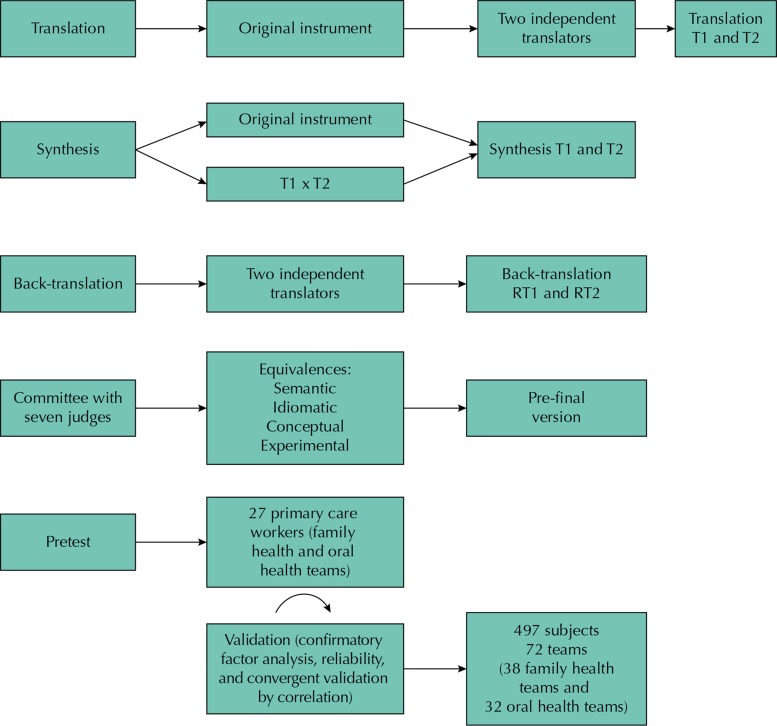
Cross-cultural adaptations and validations stages.

Methodological study with quantitative approach of cross-cultural adaptation of a instrument according to method proposed by Beaton et al.^a^, which includes the steps of cross-cultural adaptation and validation, shown in Figure 1.

### Instruments

#### Team Climate Inventory (TCI)

The instrument is composed of 38 items divided into four factors: participation in the team (12 items), support for new ideas (8 items), team goals (11 items), and task orientation (7 items). The factors are composed of statements or questions in the Likert-type scale.

The “participation in the team” factor evaluates whether the team members feel safe to exhibit their perceptions without feeling judged or censored by other team members (communication and interaction). The “support for new ideas” dimension refers to the concrete and practical support, so that the team can introduce new actions or improve the execution of activities in the workplace in response to the users’ health needs. The “team goals” factor measures how much the team has clear and defined objectives to develop and propose work methods as well as to share the perceptions of teamwork. The “task orientation” dimension evaluates both the individual and team perception about responsibility and individual and team commitment regarding the performance of tasks, in search of quality, monitoring, critical analysis, and other forms of control and analysis of the performance of health actions^1^.

#### Occupation Stress Indicator (OSI)

For the convergent validation, we used the OSI^16^ instrument, which measures occupational stress sources and their consequences for the worker from a set of situations (stressful sources) and from the individual (behavior) before the work.

The job satisfaction subscale was adapted and validated in Brazil^17^ and is composed of 22 items that assess the satisfaction of employees by the Likert-type scale.

## Study Location

The study was carried out in the city of Campinas, Sao Paulo, Brazil, as it has a structured primary healthcare network, highlighted on the national scenario as one of the pioneers in the implementation of models and strategies for health care within SUS^11^. In 2013, when the instrument was validated, the percentage of coverage of FHS^b^ was 33.6%, and, in July 2015, 53.6%. The city has representative features of large cities in the Country and has a projection for 2014-2015^c^ of more than 1.5 million people, with life quality indicators similar to those of other cities with more than 500,000 inhabitants.

The composition of the family health teams does not correspond to the classic model proposed by the Ministry of Health^9^, since it has the following professionals: one general practitioner, one pediatrician, one gynecologist-obstetrician, one nurse, four nursing assistants, four to six community health workers, one dentist, and one assistant or technician in oral health, defined as expanded reference team^11^.

The research was approved by Ethics Committee (Process 04139512.0.0000.5392) and authorized by the Municipal Secretariat of Health of Campinas. The workers who have agreed to participate in the research have signed the informed consent form.

## Method of the Cross-cultural Adaptation Stage

Two independent translations were carried out, later compared by the researchers to produce a synthesis of the similarities found. Two back-translations with independent translators who did not have contact with the original instrument and translations were also carried out.

After the translations and back-translations, we formed an expert committee (seven specialists) with fluency in English and experiences in teamwork in the primary care, health promotion, public health, human resources management, and methodology of cross-cultural adaptation. The goal of the committee was to evaluate and identify discrepancies in the translation and, thus, achieve consensus in the group of evaluators to ensure the equivalence of the adapted version to the original version of the scale. The assessment was done in two steps: individual evaluation and meeting for definition of consensus, in particular of the items in the scale that did not reach 80.0% of agreement^20^. The pretest of the adapted version was applied to two family health teams and one oral health team, selected by convenience and not included in the validation sample. The application of the instrument took place between June and July 2013.

## Method of the Validation Stage

For sample estimation, we requested in each district the following data (total) of: health centers per district, teams and complete teams, totaling 225 full teams distributed in the five districts. We considered as a complete family health team: one general practitioner, one nurse, two nursing assistants, and three community health workers and, for the oral health team, one dentist and one oral health assistant, acting in the respective team for at least six months.

The sample was distributed by conglomerates (teams) in one single stage, using implicit stratification by type of team and districts. The sampling fraction was 0.32 (73/225), and we raffled 72 teams systematically, totaling 497 professionals inserted in both teams. Due to missing data, 453 individuals were considered as valid data for the statistical analysis.

The instrument was applied in 27 (57.4%) health centers by team meeting and in 20 (42.6%) health centers by meetings with each professional, according to availability. We received refusal from six health centers, which have been replaced, and from 16 professionals (two general practitioners, one nurse, six nursing assistants, and seven community health workers).

## Statistical Analysis

Data were analyzed by structural equation modeling (SEM) via partial least square (PLS), in the SmartPLS software, version 2.0 M3. The PLS models the interrelationships between the latent variables and their indicators and has been regularly applied to confirmatory tests in researches because it converges the data on estimated parameters, when the maximum likelihood estimation cannot be found^6^.

The analysis by PLS includes the measurement model and the structural model. The measurement model is estimated using correlations between latent variables and their respective manifest variables. The structural model is estimated by the correlation between latent variables. To verify the adequacy of the model, we evaluate the following parameters:

Reliability: the traditional indicator is the Cronbach’s alpha, based on inter-correlations between the latent variables, but the composite reliability is more adequate to the PLS program, because it prioritizes the variables according to their reliabilities, while the Cronbach’s alpha is more sensitive to the number of variables in each construct^6^.

Convergent validity: assessed by Average Variance Extracted (AVE), the mean variance is extracted and measures how much manifest variables correlate positively with their respective latent variables (mean of the correlation). The literature considers there is convergent validity when the AVE value is greater or equal to 0.50^6^. When it does not reach the expected value, we need to evaluate the factor loading of the items, to delete the items that present loading below 0.50, and to estimate the model again. When the withdrawal of items with loading below 0.50 does not increase the AVE value, we need to remove the items with loading below 0.70 and estimate the model again. To assess the significance between the manifest variables and the constructs, we need to apply the t-test by the Bootstrapping (resampling) using a random resampling with 1,000 repetitions. Correlations with values > 1.96 are considered significant (5% significance level)^6^.

Discriminant validity: assessed by the cross loading (items present higher factor loadings in their respective constructs than in others) of the square root of AVE of each latent variable, comparing the value of correlation between latent variables. To have discriminating validity, the value of the square root of AVE must be larger than the value of the correlation between the constructs^6^.

We presented to the teams participating in the study, in addition to TCI, the job satisfaction scale to carry out the convergent validity by correlation. For this analysis, we used the Pearson’s correlation, with significance p < 0.001.

## Cross-cultural Adaptation – Experts Committee

In the experts’ individual evaluation, the instrument with 46 items (including title, description, and item of each factor) presented 14 items that did not reach the agreement of 80.0%. At the first meeting of the committee, doubts about the items 9, 11, and 12 of the “participation in the team” factor remained (Table 1), which were submitted for discussion with the authors of the scale.

**Table 1:** Set of items of the “participation in the team” factor that did not reach agreement at the first meeting of the experts committee.

Item	Translation 1	Translation 2	Synthesis	Equivalence problem
9. We interact frequently	*Interagimos com frequência*	*Interagimos frequentemente*	*Interagimos frequentemente*	Conceptual and experimental
11. We keep in touch with each other as a team	*Mantemo-nos em contato como uma equipe*	*Mantemo-nos em contato como equipe*	*Mantemo-nos em contato como equipe*	Idiomatic, experimental, and conceptual
12. Members of the team meet frequently to talk both formally and informally	*Os membros da equipe se encontram com frequência para conversar tanto formal quanto informalmente*	*Os membros da equipe se encontram frequentemente para conversas formais e informais*	*Os membros da equipe se encontram frequentemente para conversas formais e informais*	Conceptual

The experts pointed the semantic proximity between these items, as if they were the same statement, but with different words. In item 12, they questioned about the frequency (numbers) with which the team members meet and about the difference between formal meetings (team meetings and other activities formally carried out in the workplace) and informal meetings (such as conversations in the hallway, among others).

According to the authors of the TCI, the types of contact in the three questioned items are different from each other, which allow to identify the frequency and characteristics of the interaction of team members, as if they were a subdimension of the factor.

The experts also questioned the “team goals” factor about how much the primary care teams in Brazil work with specific and defined goals. The members of the committee considered more plausible that the teams act based on general objectives both from the regulatory plan and from the agreed in the units. The committee suggested contacting the authors about which would be the goals to be evaluated.

The author Michael West replied that in the United Kingdom the studies also find difficulties from the teams in the definition of objectives, and suggested keeping all items of the factor that has been shown to be predictive in the researches, because the teams that do not have clarity of the focus of their its activities (goals) tend to be less effective.

In a second meeting, the committee followed the authors’ guidelines and proposed to the researcher to include the items 9, 11, and 12 of the “participation in the team” factor and all items of the “team goals” factor in the interview with the participants of the pretest, to investigate their understanding, questions, and suggestions.

## Pretest

Most participants were women (23; 85.4%), with predominant workload of 36 hours, exclusive dedication in a single unit, and working time in City Hall, health unit, and team, respectively, of 9.4 years, 5.0 years, and 4.6 years. The professionals participating were: community health workers, four (14.8%); nursing auxiliaries, ten (37.0%) nurses, four (14.8%); general practitioner, two (7.4%); obstetrician-gynecologist, three (11.2%); pediatrician, two (7.4%); dentist, one (3.7%), and oral health assistant, one (3.7%). We calculated the Cronbach’s alpha with values from 0.89 to 0.92.

In evaluating the response of the participants regarding items 9, 11, and 12, they identified several types of contact between team members that ranged from the contact at lunchtime, meeting in the hallway to cases discussion, scheduled team meetings, and travels and year-end parties. Six (22.2%) participants reported that item 12 was similar to items 9 and 11 and that they considered the same type of contact when answered the instrument. We decided to keep all items in the instrument in the validation stage and to verify how the items would behave in the statistical analyses.

As to the team goals factor, 18 (66.7%) participants reported that the team goal is to offer good care to the users of the service, meeting their needs and promoting health. Oral health professionals stated general goals, such as promotion and quality of health care, but did not state clearly what would be the team goal of oral health. Only one (3.7%) participant said not having clarity of team goals, as this participant was there for less than six months. The application of the instrument took on average 14 minutes and 52 seconds.

## Analysis of the Psychometric Properties of TCI

The sample consisted of 417 (84.1%) women; average age of 42.2 years; working time in City Hall, unit, and team of, respectively, 9.5 years, 6.9 years, and 5.6 years; 420 (85.4%) professionals with workload of 36 hours; and 477 (96.4%) professionals working in a single health unit. The professional category was distributed in: obstetrician-gynecologist, 20 (4.0%); pediatrician, 35 (7.0%); oral health assistant, 36 (7.2%); dentist, 39 (7.8%); general practitioner, 41 (8.2%); nurse, 48 (9.7%); community health worker, 128 (25.8%), and nursing assistant, 150 (30.2%).

**Figure 2:**
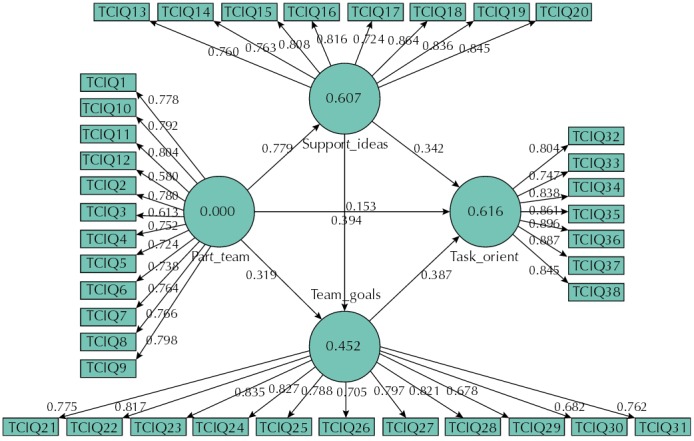
Structural model of the TCI scale.

When performing the confirmatory factor analysis, we confirmed the original model of four factors and 38 items, with factor loadings above 0.50 (Figure 2). The Cronbach’s alpha, composite reliability, and convergent validity (AVE) are presented in Table 2.

TCIQ: Team Climate Inventory Question; Task_orient: task orientation; Part_team: participation in the team; Team_goals: team goals; Support_ideas: support for new ideas

**Table 2:** Values of Cronbach’s alpha, composite reliability, and Average Variance Extracted (AVE). Campinas, SP, Southeastern Brazil, 2014.

Factors	Cronbach’s alpha	Composite reliability	AVE
Participation in the team	0.94	0.93	0.55
Support for new ideas	0.94	0.92	0.65
Team goals	0.94	0.93	0.60
Task orientation	0.94	0.93	0.71

When evaluating the results of discriminant validity, we noticed that the AVE square root of the “participation in the team” factor was lower than its correlation with the “support for new ideas” factor. In the evaluation of the factor loadings, we observed that only item 31 (*To what extent do you think other team members agree with these objectives?*) presented high factor loading in its factor (team goals) and in the “task orientation” factor. However, the square root of these two factors has not shown values below the correlation between both, which allowed to maintain the item on the scale (Table 3).

**Table 3:** Correlation between the factors and square root of the Average Variance Extracted (AVE). Campinas, SP, Southeastern Brazil, 2014.

Factors	Support for new ideas	Team goals	Task orientation	Participation in the team
Support for new ideas	**0.80**			
Team goals	0.64	**0.77**		
Task orientation	0.71	0.70	**0.84**	
Participation in the team	0.78	0.63	0.66	**0.74**

Discriminant validity presented in bold.

Another option was to evaluate the factor loading of the items in their respective latent variable, with values below 0.70, which showed that two items presented loading below 0.70 in the “participation in the team” factor (item 3: *We all influence each other* – 0.58; item 12: *Members of the team meet frequently to talk both formally and informally* – 0.61). In removing these items and estimating the model again, the AVE square root increased to 0.78, reaching the expected discriminant validity and proving that the factor measures what it intends to measure.

The alternative of exclusion of the items 3 and 12 was discussed with the authors of the TCI. The authors argued for not excluding them, since the instrument was validated in 11 countries and presented good psychometric results. Thus, we decided to keep the items 3 and 12, with a view to apply the scale in other scenarios of primary care in Brazil.

The correlation of the Portuguese version of the TCI with the job satisfaction scale was positive and weak (total TCI: 0.464; Support for new ideas: 0.417; Team goals: 0.417; Task orientation: 0.434; Participation in the team: 0.360), considering p < 0.001.

## DISCUSSION

The psychometric properties of the Portuguese version of the TCI scale were analyzed via PLS, which includes the assessment of construct reliability and validity and, in our knowledge, is the first validation of the TCI with such method.

In the construct validation, item 3 of the “participation in the team” factor (*We all influence each other*) presented factor loading below 0.70. In the version validated in Portugal^d^, this item also presented low factor loading in the exploratory factor analysis, attributed to a possible lack of semantic clarity, since the word “influence” can have been interpreted both in the positive and in the negative sense. In the studies of Greece^2^ and Netherlands^13^, in the reliability analysis between items, this item showed low correlation with other factor items, i.e., it did not contribute to the aggregate with others and, when it was excluded, the value of the Cronbach’s alpha presented significant improvement^2^.

The “team goal” factor was questioned by the experts committee, which identified possible problems as the study participants could not have clarity on the specific goals of their respective team, and maybe this could cause noise to the statistical analysis. The study carried out in Greece^2^ also shows questions regarding this factor, since the teams do not work with common goals, but with goals imposed by the management hierarchy. The authors state that the work teams, especially in the public sector, have common goals, but that are only achieved when new processes and systems in accordance with the global market are introduced^2^.

The correlation between the TCI scale and job satisfaction subscales showed a weak, but significant, correlation. This shows the need to explore the correlation between team climate and job satisfaction in the context of primary health care in Brazil in other realities, and to identify if the favorable or unfavorable climate for team work influences the worker’s satisfaction at work. The study carried out in Australia^15^ with primary care workers shows correlation between teamwork climate and job satisfaction, highlighting that the teamwork climate is a predictor of job satisfaction.

The limitations of the study refer to the scale being validated in only one city, although large and representative; to the justification of keeping items 3 and 12, even with factor loading below 0.70, and points to the need for applying the instrument in other places of the country.

The version of the teamwork climate scale adapted and validated in Brazil resulted in 38 items distributed on four factors (model proposed by the authors of the original scale), and features equivalent psychometric properties.

The validated instrument allows us to discriminate different types of teams according to the four factors analyzed and to identify areas in which the teams are stronger or more fragile and, based on the results, develop permanent education initiatives to increase the effectiveness of the teams. This deserves to be analyzed, because teamwork is a primary care guideline of SUS, which reached, in 2015, 39,686 family health teams and 22,183 oral health teams^9^.

The application of the validated scale will diagnose the teams regarding the participation of their members, the definition of goals, the team self-monitoring to achieve the expected results, and the supporting innovation before the needs of users. The teamwork climate scale can also be used in researches that assess the impact of teamwork associated with other primary care assessment tools, such as those used in the National Program for Improving Access and Quality of Primary Health Care (PMAQ-AB) and in the Primary Care Assessment Tool (PCATool).

## Footnotes

a Beaton D, Bombardier C, Guillemin F, Ferraz MB. Recommendations for the cross-cultural adaptation of the DASH & Quick DASH outcome measures. Toronto: Institute for Work & Health; 2007 [cited 18 Jun 2015]. Available from: http://dash.iwh.on.ca/system/files/X-CulturalAdaptation-2007.pdf

b Ministério da Saúde, Departamento de Atenção Básica. Histórico de cobertura da Saúde da Família. Brasília (DF): Ministério da saúde; 2015 [cited 21 Sept 2015]. Available from: http://dab.saude.gov.br/dab/portaldab/historico_cobertura_sf.php

c Instituto Brasileiro de Geografia e Estatística. Sinopse do censo demográfico 2010: Rio de Janeiro. Rio de Janeiro (RJ): IBGE; 2010 [cited 18 Jun 2015]. Available from: http://www.censo2010.ibge.gov.br/sinopse/index.php

d Cardoso CSG. Clima de equipa, inovação e liderança: convergências possíveis numa organização do sector agro-alimentar [dissertation]. Coimbra: Faculdade de Psicologia e de Ciências da Educação, Universidade de Coimbra; 2009.
