# Erratum

**DOI:** 10.1590/S01518-8787.2016050006484err

**Published:** 2016-10-26

In the article: **“Cross-cultural adaptation and validation of the teamwork climate scale”** published in the “Revista de Saúde Pública”, 2016;50:52, DOI:10.1590/S01518-8787.2016050006484, in Mariana Charantola Silva’s institution, **where it reads:** “Prefeitura Municipal de Campinas. *Secretária de Saúde.* Campinas, SP, Brasil”, **it should read:** “Prefeitura Municipal de Campinas. **Secretaria de Saúde.** Campinas, SP, Brasil”.

